# Long‐Term Safety Profile of Ruxolitinib in Chronic Myeloproliferative Neoplasms: A Comprehensive Real‐World Analysis

**DOI:** 10.1002/jha2.70152

**Published:** 2025-10-29

**Authors:** Alberto Blanco‐Sánchez, Rosa Ayala, Gonzalo Carreño‐Tarragona, Rafael Colmenares, Nieves López‐Muñoz, Adolfo Sáez, María Luisa Palacios‐Berraquero, Julia Hernández, Joaquín Martínez‐López

**Affiliations:** ^1^ Hematology Department, Hospital 12 de Octubre, CNIO Complutense University Madrid Spain; ^2^ Data Science Group Hospital 12 de Octubre Madrid Spain

**Keywords:** chronic myeloproliferative neoplasm, infection, non‐melanoma skin cancer, ruxolitinib

## Abstract

**Introduction:**

Ruxolitinib is a key therapeutic option for patients with myeloproliferative neoplasms. Its short‐term toxicity profile is well‐established, but long‐term safety data remains scarce.

**Methods:**

We aimed to evaluate toxicity associated with long‐term exposure to ruxolitinib (defined as 3 years or longer) through a dual‐cohort retrospective study combining a local cohort from Hospital 12 de Octubre with a large real‐world dataset from TriNetX database. The selected outcomes were non‐melanoma skin cancer (NMSC), other secondary malignancies and infections (zoster, urinary tract infection, pneumonia and sepsis).

**Results:**

With a median treatment time of 61.5 months in the local cohort (*n* = 36), infections were common (72.2% of patients), with occurrence of late‐onset opportunistic infections such as disseminated mycobacterial disease. A total of 19.4% of patients developed NMSC and 11.1% other secondary malignancies. In the TriNetX cohort, after propensity score matching (*n* = 2579), patients with ≥ 3 years of ruxolitinib showed a higher risk of NMSC, zoster, urinary tract infection, pneumonia and sepsis. No significant increase in other tumours was observed.

**Conclusion:**

Our findings suggest that extended ruxolitinib therapy is associated with specific long‐term risks, particularly NMSC and zoster reactivation, while not increasing the incidence of secondary malignancies. These results support continued vigilance and preventive strategies in the setting of long‐term ruxolitinib exposure.

**Trial Registration:**

The authors have confirmed clinical trial registration is not needed for this submission

## 1 Introduction

Myeloproliferative neoplasms (MPNs)—including essential thrombocythaemia (ET), polycythaemia vera (PV) and myelofibrosis (MF)—represent a group of haematological clonal disorders characterised by the excessive proliferation of myeloid cell lineages [1]. The acquisition of driver mutations in haematopoietic stem‐cells (most commonly in *JAK2*, *CALR* or *MPL*) is a key feature of their pathogenesis and diagnosis [2]. Among the treatment options available for MPNs, JAK inhibitors (JAKi) have emerged as a significant therapeutic advancement [3]. Ruxolitinib is the most broadly used drug within this pharmacological family [3].

Several clinical trials have established ruxolitinib's efficacy in providing symptomatic relief, spleen size reduction and improvement of survival rates in MF [4, 5, 6, 7]. Thus, it was approved by the FDA in 2011 [8] and has since become a frontline treatment for this condition [9, 10]. Given its effectiveness, it was later approved for patients with PV and intolerance or resistance to hydroxyurea [11, 12, 13]. Neither FDA nor EMA have approved ruxolitinib for ET, but it may offer an option for patients with intolerance or refractoriness to other therapies, especially those with a high‐symptomatic burden [14].

The short‐term toxicity profile of ruxolitinib is well‐established, primarily consisting of haematological adverse events such as anaemia and thrombocytopenia [15], and an increased risk of infections, particularly herpes zoster reactivation. The inhibition of JAK‐STAT pathway might play a relevant role in these adverse events [16]. However, the consequences of continuous immunosuppression derived from long‐term exposure are still poorly understood, since a significant proportion of patients with MF discontinue ruxolitinib therapy early [17]—approximately half of the patients ceased treatment within a 3‐year period in pivotal trials, mostly because of intolerance, suboptimal efficacy or loss of response [17].

In this sense, the potential increased risk of non‐melanoma skin cancer (NMSC) has emerged as a concern [18]. Several studies and case reports have evidenced a higher incidence of NMSC [19, 20, 21], raising questions about the need for enhanced dermatological surveillance and preventive measures in this patient population [18].

Beyond NMSC, the potential association with other secondary malignancies has been subject of debate [22, 23]. While some studies have raised concerns about the possible association with specific malignancies like lymphoproliferative neoplasms [24], most recent evidence has reported no significant increase in cancer risk overall [25, 26, 27]. The heterogeneity of findings across different studies underscores the need for a comprehensive analysis of long‐term data to identify any potential causal relationships.

Regarding the risk of infection, analysis from clinical trials and retrospective series have not shown an increased risk associated to long‐term therapy [28]. However, there are some reports of opportunistic infections as late as 9 years after ruxolitinib initiation [29].

To date, no large studies have focused on patients with long‐term exposure to ruxolitinib. As patients with MPNs often require lifelong treatment, understanding the long‐term implications of ruxolitinib therapy is crucial for optimising patient care and risk management strategies.

This paper aims to provide a comprehensive study of the long‐term safety profile of ruxolitinib in patients with MPNs. To this purpose, we analysed a cohort from our hospital and a larger real‐world population obtained from TriNetX, as described in the following section. This platform has already been used by other authors in the field of other MPN, such as chronic myeloid leukaemia [30, 31] and MF [32].

## 2 Material and Methods

### 2.1 Study Population and Design

We performed a retrospective study of patients with MPN treated with ruxolitinib for a minimum of 3 years between 2011 and 2024 at Hospital Universitario 12 de Octubre. We further analysed a large cohort of patients with MPN stratified according to the exposure to ruxolitinib between using TriNetX electronic medical records (EMRs) [33]. This is a global federated clinical research database that enables access to real‐time, de‐identified EMRs from a network of healthcare organisations. This network included 144 healthcare organisations worldwide at the time of analysis.

No definition of long‐term exposure to ruxolitinib has been made in literature. However, based on the median time of treatment reported in pivotal trials, a cut‐off of 3 years was set for this category [17].

Patients with MF, PV or ET were identified using the International Classification of Diseases, 10th Revision (ICD‐10) codes D75.81, D45 and D47.3, respectively. Patients were initially divided into three cohorts: those receiving ruxolitinib for < 3 years, for ≥ 3 years and individuals treated with a non‐JAKi cytoreductive agent (hydroxyurea, anagrelide or interferon alfa‐2a). Patients receiving other JAKi (fedratinib, momelotinib and pacritinib) or allogeneic transplantation were excluded.

In order to ensure homogeneity across cohorts and to avoid immortal time bias, only patients with a minimum follow‐up of 3 years were included. Even after applying this selection criterion, the follow‐up time of the short‐term users was too disparate to establish an adequate comparison, so this cohort was not further analysed (Supporting Information).

Initiation of therapy was defined as the index date (either ruxolinitib or cytoreductive agent for each group). The study excluded patients that met the index date more than 20 years ago. The follow‐up period was set at a maximum of 3300 days from the index date (approximately 9 years). The outcomes assessed were NMSC, other solid tumours, lymphoma, zoster infection, urinary tract infection, pneumonia and sepsis.

### 2.2 Statistics

To minimise confounding factors, the cohorts were balanced using 1:1 propensity score matching performed through the TriNetX platform, as previously described [34]. Propensity scores were estimated using logistic regression based on selected covariates, including age, sex, white race, tobacco use, actinic keratosis, prior NMSC, overweight and obesity and type of MPN (MF, PV or ET). The platform uses greedy nearest‐neighbour approach with a caliper of 0.1 pooled standard deviations [34]. Other possible confounding factors such as international prognostic scoring systems, mutational status or splenomegaly could not be included due to lack of information from the data source.

Frequencies were calculated as percentages for qualitative variables and a median and interquartile range for quantitative variables. Descriptive statistics of the local cohort was conducted using R 4.3.2 (version 4.3.2).

Statistical analyses for the TriNetX cohort were performed using the built‐in analytics of the platform in April 2025. The incidence of outcomes was analysed as event‐free survival. Differences in survival were assessed through Kaplan–Meier analysis with the Log‐Rank test. Hazard ratios and their associated 95% confidence intervals were also calculated. Statistical significance was set at *p* < 0.05.

#### 2.2.1 Ethics

This study was approved by the ethics committee of the Hospital Universitario 12 de Octubre and conducted in accordance with the Declaration of Helsinki.

## 3 Results

A total of 36 patients were treated in our centre, with a median age of 66.5 years (57.8–73.5) at the beginning of therapy and median time of exposure was 61.5 months (range 36–151 months). A total of 11 patients (30.6%) had primary MF, 18 (50%) secondary MF, 5 (13.9%) PV and 2 (5.5%) had ET. A total of 27 patients (75%) had been previously exposed to hydroxyurea and 12 (33.3%) to two or more agents (anagrelide and/or interferon). Table 1 summarises the main characteristics of this cohort.

**TABLE 1:** Baseline characteristics of the local cohort.

	Median/absolute frequency	IQR/relative frequency
Age (years)	66.5	57.8–73.5
Male/female	13/23	36.1%/63.9%

Diagnosis		
PMF	11	30.6%
SMF	18	50%
PV	5	13.9%
ET	2	5.5%

DIPSS (only for MF, *n* = 29)		
Low	5	13.9%
Intermediate‐1	12	33.3%
Intermediate‐2	12	33.3%
High	7	19.4%

Previous treatment		
Hydroxyurea	27	50%
None	8	22.2%
Anagrelide	8	22.2%
IFN	7	19.4%

Abbreviations: DIPSS, Dynamic International Prognostic scoring System; ET, essential thrombocythaemia; IFN, interferon alfa‐2a; IQR, interquartile range; PMF, primary myelofibrosis; PV, polycythaemia vera; SMF, secondary myelofibrosis.

**FIGURE 1:**
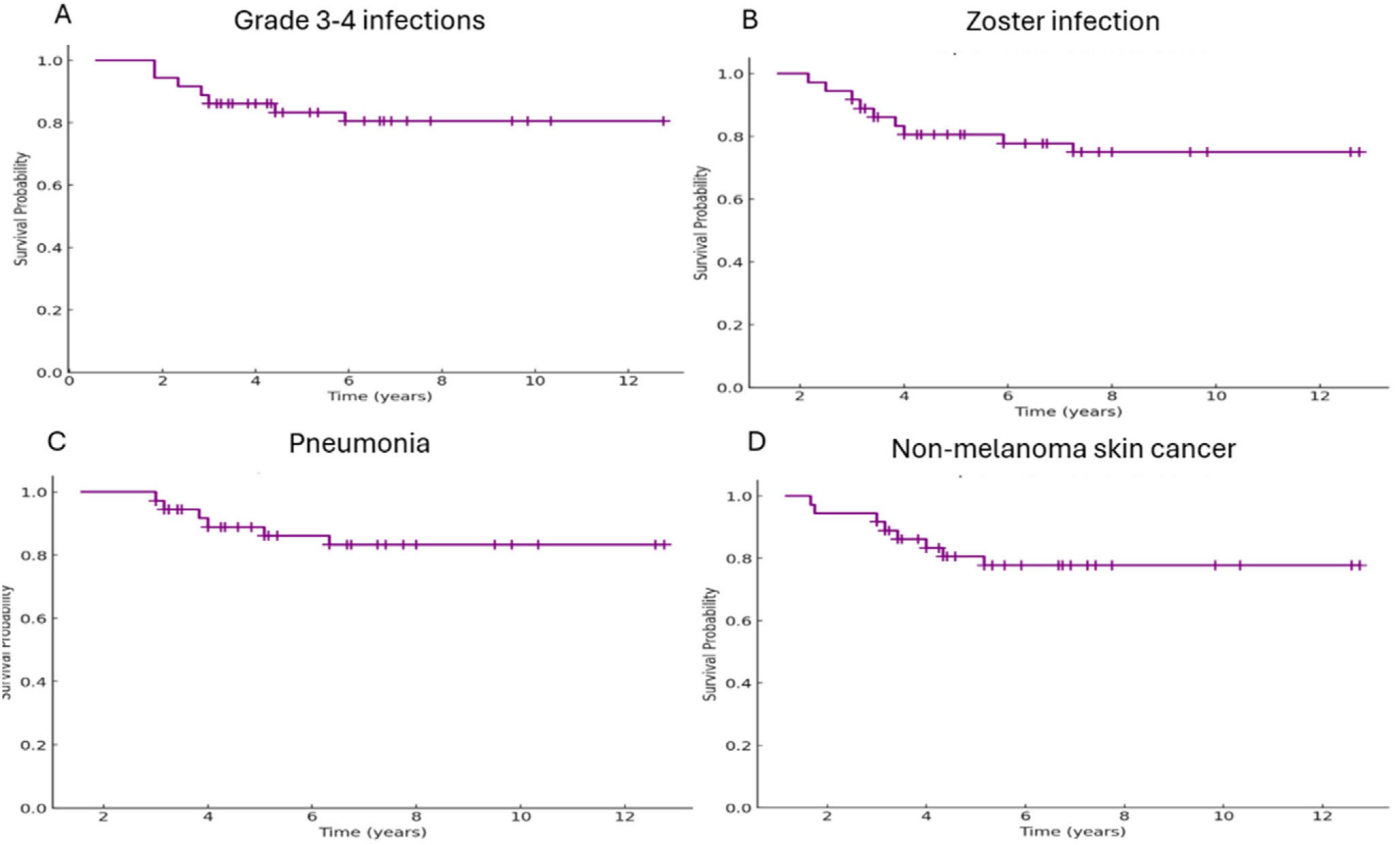
Kaplan‐Meier curves for adverse events in the local cohort.

During follow‐up, 26 patients (72.2%) presented an infection of any kind. The most frequent infections were upper respiratory tract infection (23, 63.9%), herpes zoster infection (7, 19.4%), urinary tract infection (6, 16.7%) and pneumonia (6, 16.7%). Nine patients (27.3%) presented Grade 3–4 infections (Figure 1A). Of note, several patients had late‐occurring infections: one patient had an *Actinomyces* infection and three had disseminated mycobacterial infections, as late as 6.8 years after ruxolitinib initiation. There were no hepatitis B reactivations, *Pneumocystis* or invasive fungal infections.

Most infections by herpes zoster (71.4%) occurred during the first 3 years of treatment, although an episode was reported as late as 5.5 years after ruxolitinib initiation (Figure 1B). Regarding pneumonia, most cases took place beyond 3 years of exposure (Figure 1C), but this was clearly impacted by SARS‐CoV2 pandemics, since four out of six episodes were due to this pathogen.

With respect to secondary neoplasms, seven patients (19.4%) developed NMSC with a median time to the first NMSC occurrence of 3.25 years (range 1.2–4.7). Three individuals had had NMSC prior to ruxolitinib initiation, and all of them had been previously exposed to hydroxyurea. Four patients presented with squamous cell cancer, one with basal‐cell carcinoma and two with both types. The event free survival curves for the aforementioned adverse events appears to reach a plateau (Figure 1).

Four patients (11.1%) developed a secondary malignancy other than NMSC under treatment with ruxolitinib: endometrial cancer, epidermoid parotid cancer, prostate cancer and breast cancer. None of the 36 patients were diagnosed with lymphoma during follow‐up.

Regarding the TriNetX network, a total of 12,883 patients with MPN were identified. A total of 1368 patients received ruxolitinib treatment for ≥ 3 years, with a median time of exposure to the drug of 66.3 months (IQR 49.3–88.8). Furthermore, 11,515 were treated without a JAKi. Baseline characteristics of both cohorts are depicted in Table 2.

**TABLE 2:** Baseline characteristics of the cohorts from TriNetX before matching.

	Exposure ≥ 3y	No exposure	*p*

Cohort size, *n*	1368	11,515	
Mean age (SD), years	65.6 (12.2)	67.8 (14)	< 0.001

Sex			
Male, *n* (%)	576 (42.1)	4249 (38.1)	0.0044
Female, *n* (%)	714 (52.2)	6467 (58)	< 0.001
Unknown, *n* (%)	78 (5.7)	418 (3.9)	NS

Type of MPN			
Myelofibrosis	420 (30.7)	216 (1.9)	< 0.001
Polycythaemia vera	616 (45)	2998 (26)	< 0.001
ET	332 (24.3)	6160 (53.5)	< 0.001
Unknown	0	2141 (18.6)	< 0.001

Median FU (IQR), years	5.8 (3.2)	5.8 (4.6)	NS
Race			
White, *n* (%)	984 (71.9)	23,229 (65.3)	< 0.001
Black or African American, *n* (%)	73 (5.3)	4229 (11.9)	< 0.001
Asian, *n* (%)	68 (5)	1291 (3.6)	NS
Other or unknown, *n* (%)	243 (17.8)	6848 (19.2)	NS

Prior hydroxyurea	556 (41)		
Hydroxyurea at index		10,398 (90.3)	
Anagrelide at index		933 (8.1)	
Interferon at index		184 (1.6)	

Comorbidities			
Tobacco use, *n* (%)	85 (6)	949 (9)	0.006
Overweight and obesity, *n* (%)	94 (7)	999 (9)	0.022
Actinic keratosis, *n* (%)	111 (8)	623 (6)	< 0.001
Prior NMSC, *n* (%)	110 (8)	571 (5)	< 0.001

Abbreviations: ET, essential thrombocythaemia; FU, follow‐up; MPN, myeloproliferative neoplasms; NMSC, non‐melanoma skin cancer; NS, non‐significant; SD, standard deviation.

**FIGURE 2:**
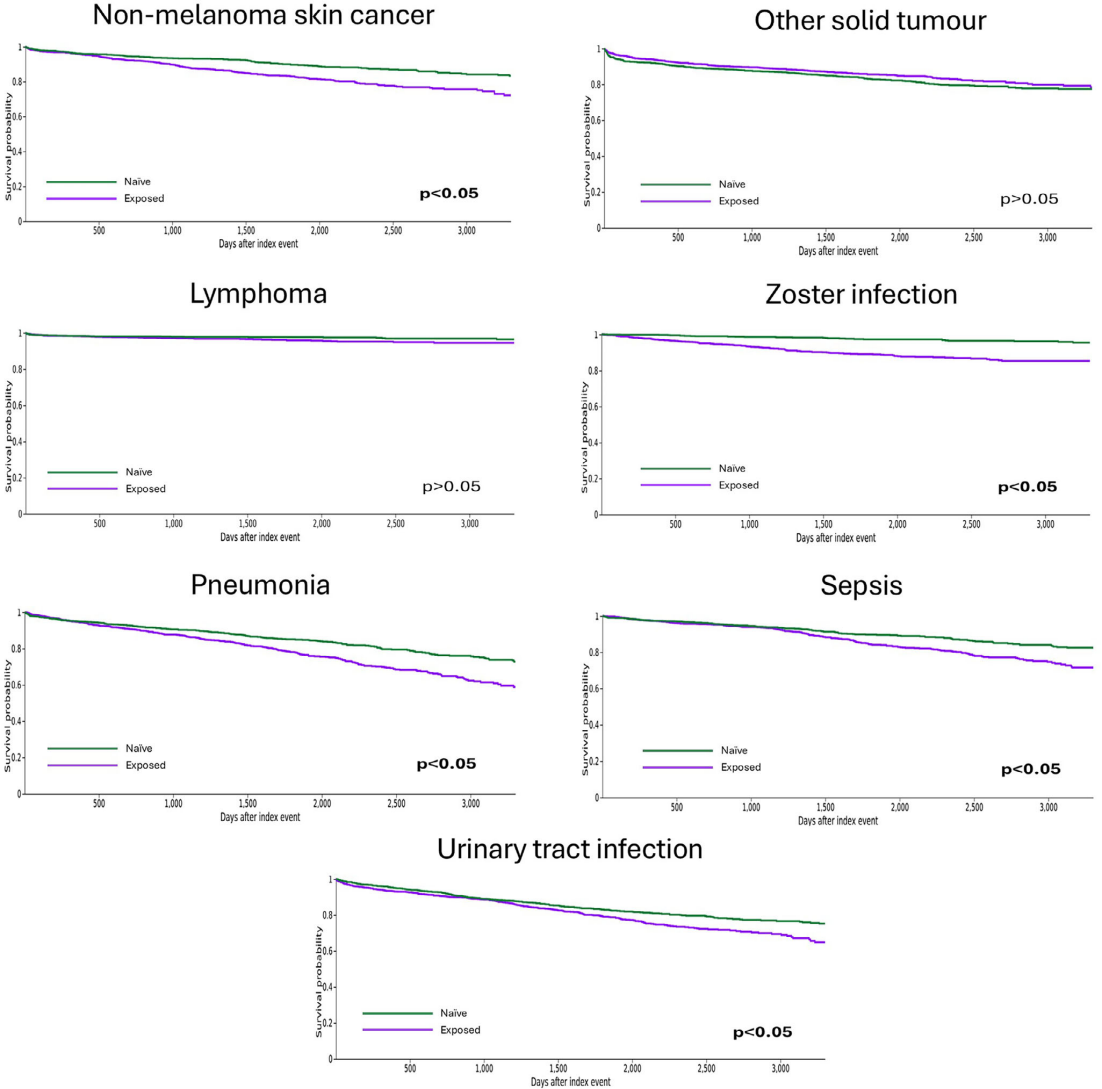
Kaplan‐Meier curves for adverse events in the TriNetX cohorts.

After propensity score matching, patients with long‐term exposure showed a higher risk of NMSC compared to ruxolitinib naïve individuals (HR 1.7, 1.3–2) (Figure 2). The distribution of histological subtypes per group was squamous (18.3% and 15.3%, *p* > 0.05), BCC (29.3% and 29.6%, *p* > 0.05) and unknown histology (52.4% and 55.1%, *p* > 0.05). There were no significant differences in the incidence of other solid tumours or lymphoproliferative neoplasms across groups (Table 3).

**TABLE 3:** Hazard ratios comparing free‐event survival between cohorts from TriNetX for each outcome.

≥ 3y vs. no exposure

Adverse event	HR	95%CI
NMSC	1.67	1.35–2.08
Other solid tumour	0.92	0.75–1.13
Lymphoma	1.62	1.00–2.61
Herpes Zoster	3.29	2.33–4.66
Pneumonia	1.51	1.26–1.79
Urinary tract infection	1.33	1.11–1.60
Sepsis	1.45	1.17–1.80

Abbreviations: 95%CI, 95% confidence interval; HR, hazard ratio; NMSC, non‐melanoma skin cancer.

Overall, risk of infection was higher in long‐term ruxolitinib users when compared to ruxolitinib‐naïve users. There was a remarkably higher risk of zoster infection (HR 3.3, 2.3–4.7). Association with pneumonia (HR 1.5, 1.3–1.8), urinary tract infection (1.3, 1.1–14) and sepsis (1.5, 1.2–1.8) was also found, although weak. There were a few cases of infection by mycobacteria, *Pneumocystis jirovecii* or *Cryptococcus neoformans* (less than 10 patients per group for each aetiology).

## 4 Discussion

To our knowledge, this is the first study specifically addressing the safety of long‐term ruxolitinib treatment in patients with MPN, defined as an exposure to the drug of ≥ 3 years. We assessed a local cohort from our institution and a large population dataset from TriNetX, a global collaborative research network that has already yielded valuable insights into this field [32]. Our findings indicate a significantly higher risk of NMSC, zoster infection, pneumonia, sepsis and urinary tract infection in long‐term treated patients, compared to ruxolitinib‐naïve patients.

Our work adds to the large body of data pointing towards an increased risk of NMSC secondary to ruxolitinib exposure. In the 5‐year update from the placebo‐controlled COMFORT‐I trial, no difference in the rate of NMSC between JAKi exposed and JAKi naïve patients was reported [6]. However, long‐term reports from the COMFORT‐II trial demonstrated a notable 17.1% incidence of NMSC in ruxolitinib‐treated patients [7]. Similarly, this was the most frequent secondary malignancy reported in the JUMP trial [4], and in a large pharmacovigilance study of 870 JAKi‐related adverse events [19]. Several studies have since confirmed this association and described distinct features of these treatment‐associated skin cancers, which typically display a more aggressive behaviour [20, 21, 35].

In a multicentre retrospective study of patients who developed NMSC after ruxolitinib, Rampotas et al. [35] identified as co‐occurring risk factors a prior history of skin cancer in 37.9% of cases and prior hydroxyurea use in nearly 70%. In their work, the predominant histology was squamous cell carcinoma (57.5%), consistent with our local cohort findings (57.1%). This is in contrast with the histological distribution of NMSC in the general population, where basal cell carcinoma is the most frequent NMSC. We were unable to confirm this in the TriNetX dataset due to data limitations.

There is currently no consensus on the optimal approach for NMSC prevention in patients receiving JAKi. An earlier version of the NCCN guidelines [10] recommended annual dermatologic examinations, while the British Society of Haematology guidelines suggest performing skin surveillance in patients with actinic keratosis or a history of skin cancer [9]. Based on our results after matching for these risk factors, it would be reasonable to include long‐term users as an especially high‐risk population for skin cancer, and perform skin surveillance accordingly.

Patients with MPN have shown an inherent higher risk of lymphoid neoplasms [36]. In 2018, Porpaczy et al. suggested an increased incidence of B‐cell lymphoma amongst patients receiving ruxolitinib, with a striking 16‐fold risk, and proposed a biological explanation based on murine models [24]. However, larger cohort analyses failed to replicate this association, attributing the initial findings to limited sample size and follow‐up [37].

Our study did not demonstrate a statistically significant association between ruxolitinib and lymphoma risk; however, the hazard ratio was elevated and the confidence interval narrowly included 1.0. We cannot exclude a true association that might be found with a larger cohort or longer follow‐up.

Previous studies have also suggested a higher risk of other solid tumours in patients with MPN [38]. However, large retrospective studies [26, 27] have not demonstrated an increased risk attributable to ruxolitinib, although their median follow‐up periods (30 and 24 months, respectively) might have been insufficient to capture such adverse events that typically develop after more prolonged periods of time. Our study, with its extended follow‐up, adds further support to the long‐term safety of ruxolitinib regarding secondary malignancies.

Besides secondary malignancies, evidence also suggests an increased risk of infections and infection‐related mortality in MPN patients compared to the general population, even in the absence of treatment [39, 40]. In a large Swedish population‐based study, Landtblom et al. [40] found a two‐fold higher risk of severe infections, irrespective of treatment status (cytoreduction or observation). The infection risk was higher in patients with MF and seemed stable over the disease course.

Herpes zoster reactivation accounts for a great percentage of ruxolitinib‐related infections [16, 41]. For this reason, primary prophylaxis with a non‐live shingles vaccine before initiation of JAKi or secondary prophylaxis with antiviral drugs may be considered [9, 10]. Our findings confirm that zoster reactivation occurs after prolonged treatment, and its rate of occurrence does not decline over time. In line with our local cohort findings, Te Linde et al. [42] showed an apparent plateau in the incidence of this complication after 5 years of treatment. However, this plateau was not observed in our larger TriNetX cohort analysis.

Beyond zoster, our analysis showed an increased rate of urinary tract infections and serious infections such as pneumonia and sepsis. However, this association should be interpreted cautiously. Polverelli et al. [43] reported a significantly higher risk of infection in patients with advanced‐stage disease, defined by high/intermediate‐2 IPSS risk and splenomegaly. These patients are more prone to receive ruxolitinib rather than non‐JAKi drugs, but we could not balance groups according to these features.

Turning to opportunistic infections, some reports have raised concerns about particular pathogens such as mycobacteria [44], histoplasma [45], *Cryptococcus* or hepatitis B [46]. The low incidence of these infections in our study confirms that these are uncommon, although careful monitoring for additional risk factors and early signs of infection is reasonable [9, 10].

The advent of novel JAKi—including fedratinib, momelotinib and pacritinib—has expanded treatment options for MF [47]. On one hand, the differential toxicity profile can favour the choice of momelotinib or pacritinib in cases where baseline cytopenias is present. On the other hand, JAKi switching has shown efficacy after frontline therapy failure [47]. Nevertheless, there is limited data on potential differences on their impact on NMSC or infection rates, despite existence of some comparative studies [48, 49].

A previous retrospective study using propensity score matching found no difference in infection rates between ruxolitinib‐treated and naïve patients [50], but the low incidence of secondary malignancies precluded achieving definitive conclusions on this respect [50]. The present study offers a propensity score‐matched analysis of a large cohort with substantial long‐term follow‐up, providing unique data on long‐term exposure and robust evidence regarding its toxicity profile.

This study has several limitations. We acknowledge that the limited size of our local cohort restricts the generalisability of its findings. For this reason we complemented the analysis with the larger cohort from TriNetX. Its retrospective design may introduce selection and information biases. Secondly, the use of electronic health records from multiple centres could lead to inconsistencies in coding and potential misclassification of diagnoses or adverse events. Although we applied propensity score matching to control for several confounders, residual confounding cannot be entirely excluded.

Moreover, data on treatment adherence, dosage adjustments and the specific reasons for ruxolitinib discontinuation were not consistently available. Important disease‐related variables such as IPSS, splenomegaly and cumulative dose are lacking due to the network characteristics.

Lastly, we could not include a group with short‐term exposure ( < 3 years) because of the significant shorter time of follow‐up. This could be explained because of early discontinuation due to disease progression, death or allogeneic transplantation.

This real‐world study provides a robust analysis of the long‐term safety profile of ruxolitinib in patients with MPN, particularly those with ≥ 3 years of exposure. The extensive data available through the TriNetX network allowed us to assemble a large sample of this otherwise underrepresented subgroup. Our results confirm an increased risk of NMSC and infections with prolonged use, while ruling out significant associations with other malignancies. This underlines the need for an adequate monitoring of these patients even after prolonged treatment periods, with special attention on skin surveillance and infection prophylaxis.

## Author Contributions

A.B.‐S. and J.M.‐L. conceived and designed the study. A.B.‐S. collected the data. A.B.‐S. and J.H. analysed and curated the data. A.B.‐S. prepared the initial manuscript draft. J.M.‐L., R.A., G.C.‐T. and R.C. guided the analysis and oriented the manuscript. N.L.‐M. and A.S. gave clinical. M.L.P.‐B. revised the manuscript for language accuracy and clarity. All authors reviewed and corrected the final manuscript.

## Ethics Statement

This study was approved by the ethics committee of the Hospital Universitario 12 de Octubre and conducted in accordance with the Declaration of Helsinki.

## Consent

Obtaining written consent from participants from TriNetX dataset was considered unnecessary, as it offers de‐identified information. The ethics committee of the Hospital Universitario 12 de Octubre granted TriNetX a waiver for informed consent, since the platform aggregates counts and statistical summaries of de‐identified data. The rest of the participants provided their written informed consent to participate in this study.

## Conflicts of Interest

The authors declare no conflicts of interest.

## Supporting information




**Supplementary Table**: Comparison of the baseline characteristics of two cohorts before matching: patients with exposure of ≥3 years versus <3years.

## Data Availability

The data from the local cohort that support the findings of this study are available from the corresponding author upon reasonable request. The rest of the data that support the findings of this study are available from TriNetX, LLC but third‐party restrictions apply to the availability of these data. The data were used under license for this study with restrictions that do not allow for the data to be redistributed or made publicly available. However, for accredited researchers, the TriNetX data is available for licensing at TriNetX, LLC. Data access may require a data‐sharing agreement and may incur data access fees.

## References

[1] J. L. Spivak , “Myeloproliferative Neoplasms,” New England Journal of Medicine 376, no. 22 (2017): 2168–2181. 28564565 10.1056/NEJMra1406186

[2] G. Greenfield , M. F. Mcmullin , and K. Mills , “Molecular Pathogenesis of the Myeloproliferative Neoplasms,” Journal of Hematology & Oncology 14, no. 1 (2021): 103. 34193229 10.1186/s13045-021-01116-z PMC8246678

[3] G. G. Loscocco and A. M. Vannucchi , “Role of JAK Inhibitors in Myeloproliferative Neoplasms: Current Point of View and Perspectives,” International Journal of Hematology 115, no. 5 (2022): 626–644. 35352288 10.1007/s12185-022-03335-7

[4] H. K. Al‐Ali , M. Griesshammer , L. Foltz , et al., “Primary Analysis of JUMP, a Phase 3b, Expanded‐Access Study Evaluating the Safety and Efficacy of Ruxolitinib in Patients With Myelofibrosis, Including Those With Low Platelet Counts,” British Journal of Haematology 189, no. 5 (2020): 888–903. 32017044 10.1111/bjh.16462

[5] A. M. Vannucchi , P. A. W. Te Boekhorst , C. N. Harrison , et al., “EXPAND, a Dose‐Finding Study of Ruxolitinib in Patients With Myelofibrosis and Low Platelet Counts: 48‐Week Follow‐Up Analysis,” Haematologica 104, no. 5 (2019): 947–954. 30442723 10.3324/haematol.2018.204602 PMC6518918

[6] S. Verstovsek , R. A. Mesa , J. Gotlib , et al., “Long‐Term Treatment With Ruxolitinib for Patients With Myelofibrosis: 5‐Year Update From the Randomized, Double‐Blind, Placebo‐Controlled, Phase 3 COMFORT‐I Trial,” Journal of Hematology & Oncology 10, no. 1 (2017): 55. 28228106 10.1186/s13045-017-0417-z PMC5322633

[7] C. N. Harrison , A. M. Vannucchi , J.‐J. Kiladjian , et al., “Long‐Term Findings From COMFORT‐II, a Phase 3 Study of Ruxolitinib vs Best Available Therapy for Myelofibrosis,” Leukemia 30, no. 8 (2016): 1701–1707. 27211272 10.1038/leu.2016.148 PMC5399157

[8] J. Mascarenhas and R. Hoffman , “Ruxolitinib: The First FDA Approved Therapy for the Treatment of Myelofibrosis,” Clinical Cancer Research 18, no. 11 (2012): 3008–3014. 22474318 10.1158/1078-0432.CCR-11-3145

[9] D. P. Mclornan , B. Psaila , J. Ewing , et al., “The Management of Myelofibrosis: A British Society for Haematology Guideline,” British Journal of Haematology 204, no. 1 (2024): 136–150. 38037886 10.1111/bjh.19186

[10] A. T. Gerds , J. Gotlib , H. Ali , et al., “Myeloproliferative Neoplasms, Version 3.2022, NCCN Clinical Practice Guidelines in Oncology,” Journal of the National Comprehensive Cancer Network 20, no. 9 (2022): 1033–1062. 36075392 10.6004/jnccn.2022.0046

[11] L. A. Raedler , “Jakafi (Ruxolitinib): First FDA‐Approved Medication for the Treatment of Patients With Polycythemia Vera,” American Health & Drug Benefits 8 (2015): 75–79. 26629270 PMC4665047

[12] C. N. Harrison , J. Nangalia , R. Boucher , et al., “Ruxolitinib Versus Best Available Therapy for Polycythemia Vera Intolerant or Resistant to Hydroxycarbamide in a Randomized Trial,” Journal of Clinical Oncology 41, no. 19 (2023): 3534–3544. 37126762 10.1200/JCO.22.01935 PMC10306428

[13] A. M. Vannucchi , J. J. Kiladjian , M. Griesshammer , et al., “Ruxolitinib Versus Standard Therapy for the Treatment of Polycythemia Vera,” New England Journal of Medicine 372, no. 5 (2015):426–435. 25629741 10.1056/NEJMoa1409002 PMC4358820

[14] A. Gunawan , P. Harrington , N. Garcia‐Curto , D. Mclornan , D. Radia , and C. Harrison , “Ruxolitinib for the Treatment of Essential Thrombocythemia,” Hemasphere 2, no. 4 (2018): e56. 31723782 10.1097/HS9.0000000000000056 PMC6746005

[15] S. Verstovsek , J. Gotlib , V. Gupta , et al., “Management of Cytopenias in Patients With Myelofibrosis Treated With Ruxolitinib and Effect of Dose Modifications on Efficacy Outcomes,” OncoTargets and Therapy 7 (2013): 13–21. 24368888 10.2147/OTT.S53348 PMC3869911

[16] F. Lussana , M. Cattaneo , A. Rambaldi , and A. Squizzato , “Ruxolitinib‐Associated Infections: A Systematic Review and Meta‐Analysis,” American Journal of Hematology 93, no. 3 (2018): 339–347. 29150886 10.1002/ajh.24976

[17] C. N. Harrison , N. Schaap , and R. A. Mesa , “Management of Myelofibrosis After Ruxolitinib Failure,” Annals of Hematology 99, no. 6 (2020): 1177–1191. 32198525 10.1007/s00277-020-04002-9 PMC7237516

[18] A. Rampotas , R. Hargreaves , and D. P. Mclornan , “Challenges of Diagnosing and Managing Pre‐Fibrotic Myelofibrosis: A Case‐Based and Practical Approach,” Best Practice & Research Clinical Haematology 35, no. 2 (2022): 101378. 36333067 10.1016/j.beha.2022.101378

[19] C. Jalles , M. Lepelley , S. Mouret , J. Charles , M.‐T. Leccia , and S. Trabelsi , “Skin Cancers Under Janus Kinase Inhibitors: A World Health Organization Drug Safety Database Analysis,” Therapies 77, no. 6 (2022): 649–656. 10.1016/j.therap.2022.04.005 35710462

[20] J. Q. Lin , S. Q. Li , S. Li , et al., “A 10‐Year Retrospective Cohort Study of Ruxolitinib and Association With Nonmelanoma Skin Cancer in Patients With Polycythemia Vera and Myelofibrosis,” Journal of the American Academy of Dermatology 86, no. 2 (2022): 339–344. 34648874 10.1016/j.jaad.2021.10.004

[21] A. B. Blechman , C. E. Cabell , C. H. Weinberger , et al., “Aggressive Skin Cancers Occurring in Patients Treated With the Janus Kinase Inhibitor Ruxolitinib,” Journal of Drugs in Dermatology 16, no. 5 (2017): 508–511. 28628689

[22] S. Verstovsek , R. A. Mesa , R. A. Livingston , W. Hu , and J. Mascarenhas , “Ten Years of Treatment With Ruxolitinib for Myelofibrosis: A Review of Safety,” Journal of Hematology & Oncology 16, no. 1 (2023): 82. 37501130 10.1186/s13045-023-01471-z PMC10373260

[23] B. Mora , E. Rumi , P. Guglielmelli , et al., “Second Primary Malignancies in Postpolycythemia Vera and Postessential Thrombocythemia Myelofibrosis: A Study on 2233 Patients,” Cancer Medicine 8, no. 9 (2019): 4089–4092. 31173472 10.1002/cam4.2107 PMC6675726

[24] E. Porpaczy , S. Tripolt , A. Hoelbl‐Kovacic , et al., “Aggressive B‐Cell Lymphomas in Patients With Myelofibrosis Receiving JAK1/2 Inhibitor Therapy,” Blood 132, no. 7 (2018): 694–706. 29907599 10.1182/blood-2017-10-810739 PMC7115916

[25] R. Sekhri , P. Sadjadian , T. Becker , et al., “Ruxolitinib‐Treated Polycythemia Vera Patients and Their Risk of Secondary Malignancies,” Annals of Hematology 100, no. 11 (2021): 2707–2716. 34462786 10.1007/s00277-021-04647-0 PMC8510903

[26] F. Barraco , R. Greil , R. Herbrecht , et al., “Real‐World Non‐Interventional Long‐Term Post‐Authorisation Safety Study of Ruxolitinib in Myelofibrosis,” British Journal of Haematology 191, no. 5 (2020): 764–774. 32583458 10.1111/bjh.16729

[27] M. Maffioli , T. Giorgino , B. Mora , et al., “Second Primary Malignancies in Ruxolitinib‐Treated Myelofibrosis: Real‐World Evidence From 219 Consecutive Patients,” Blood Advances 3, no. 21 (2019): 3196–3200. 31698448 10.1182/bloodadvances.2019000646 PMC6855128

[28] D. Cattaneo and A. Iurlo , “Immune Dysregulation and Infectious Complications in MPN Patients Treated With JAK Inhibitors,” Frontiers in Immunology 12 (2021): 750346. 34867980 10.3389/fimmu.2021.750346 PMC8639501

[29] A. Ritter , N. Kensey , J. Higgs , and H. Zainah , “Cavitary Lung Lesions Caused by *Pneumocystis jirovecii* in a Patient With Myelofibrosis on Ruxolitinib,” BMJ Case Reports 17, no. 8 (2024): e258468. 10.1136/bcr-2023-258468 PMC11409334 39214573

[30] A. Sanz , R. Ayala , G. Hernández , et al., “Outcomes and Patterns of Treatment in Chronic Myeloid Leukemia, a Global Perspective Based on a Real‐World Data Global Network,” Blood Cancer Journal 12, no. 6 (2022): 94. 35750670 10.1038/s41408-022-00692-8 PMC9232604

[31] R. A. B. Nunes , P. D. M. M. Neves , L. M. A. da Costa , et al., “Five‐Year Cardiovascular Outcomes in Patients With Chronic Myeloid Leukemia Treated With Imatinib, Dasatinib, or Nilotinib: A Cohort Study Using Data From a Large Multinational Collaborative Network,” Frontiers in Cardiovascular Medicine 10 (2023): 888366. 36824461 10.3389/fcvm.2023.888366 PMC9941183

[32] M. Kappenstein and N. Von Bubnoff , “Real‐World Electronic Medical Records Data Identify Risk Factors for Myelofibrosis and Can Be Used to Validate Established Prognostic Scores,” Cancers 16, no. 7 (2024): 1416. 38611094 10.3390/cancers16071416 PMC11011132

[33] TriNetX: Real‐world data for the life sciences and healthcare, accessed April 22, 2025, https://trinetx.com/.

[34] M. Taquet , S. Luciano , J. R. Geddes , and P. J. Harrison , “Bidirectional Associations Between COVID‐19 and Psychiatric Disorder: Retrospective Cohort Studies of 62 354 COVID‐19 Cases in the USA,” Lancet Psychiatry 8, no. 2 (2021): 130–140. 33181098 10.1016/S2215-0366(20)30462-4 PMC7820108

[35] A. Rampotas , L. Carter‐Brzezinski , T. C. P. Somervaille , et al., “Outcomes and Characteristics of Nonmelanoma Skin Cancers in Patients With Myeloproliferative Neoplasms on Ruxolitinib,” Blood 143, no. 2 (2024): 178–182. 37963262 10.1182/blood.2023022345

[36] E. Rumi , F. Passamonti , C. Elena , et al., “Increased Risk of Lymphoid Neoplasm in Patients With Myeloproliferative Neoplasm: A Study of 1,915 Patients,” Haematologica 96, no. 3 (2011): 454–458. 21109692 10.3324/haematol.2010.033779 PMC3046278

[37] N. Pemmaraju , H. Kantarjian , L. Nastoupil , et al., “Characteristics of Patients With Myeloproliferative Neoplasms With Lymphoma, With or Without JAK Inhibitor Therapy,” Blood 133, no. 21 (2019): 2348–2351. 30796023 10.1182/blood-2019-01-897637 PMC6634962

[38] A. R. Landtblom , H. Bower , T. M.‐L. Andersson , et al., “Second Malignancies in Patients With Myeloproliferative Neoplasms: A Population‐Based Cohort Study of 9379 Patients,” Leukemia 32, no. 10 (2018): 2203–2210. 29535425 10.1038/s41375-018-0027-y PMC7552081

[39] M. Hultcrantz , S. R. Wilkes , S. Y. Kristinsson , et al., “Risk and Cause of Death in Patients Diagnosed With Myeloproliferative Neoplasms in Sweden Between 1973 and 2005: A Population‐Based Study,” Journal of Clinical Oncology 33, no. 20 (2015): 2288–2295. 26033810 10.1200/JCO.2014.57.6652

[40] A. R. Landtblom , T. M.‐L. Andersson , P. W. Dickman , et al., “Risk of Infections in Patients With Myeloproliferative Neoplasms—A Population‐Based Cohort Study of 8363 Patients,” Leukemia 35, no. 2 (2021): 476–484. 32546727 10.1038/s41375-020-0909-7 PMC7738400

[41] Q. Luo , Z. Xiao , and L. Peng , “Effects of Ruxolitinib on Infection in Patients With Myeloproliferative Neoplasm: A Meta‐Analysis,” Hematology 26, no. 1 (2021): 663–669. 34493151 10.1080/16078454.2021.1967256

[42] E. Te Linde , L. J. E. Boots , L. G. M. Daenen , M. A. De Witte , and A. H. W. Bruns , “High Incidence of Herpes Zoster in Patients Using Ruxolitinib for Myeloproliferative Neoplasms: Need for Prophylaxis,” Hemasphere 6, no. 11 (2022): e793. 36325270 10.1097/HS9.0000000000000793 PMC9619234

[43] N. Polverelli , M. Breccia , G. Benevolo , et al., “Risk Factors for Infections in Myelofibrosis: Role of Disease Status and Treatment. A Multicenter Study of 507 Patients,” American Journal of Hematology 92, no. 1 (2017): 37–41. 27701770 10.1002/ajh.24572

[44] Y. Peng , L. Meng , X. Hu , Z. Han , and Z. Hong , “Tuberculosis in Patients With Primary Myelofibrosis During Ruxolitinib Therapy: Case Series and Literature Review,” Infection and Drug Resistance 13 (2020): 3309–3316. 33061478 10.2147/IDR.S267997 PMC7532060

[45] C.‐Y. Chiu , T. John , T. Matsuo , et al., “Disseminated Histoplasmosis in a Patient With Myelofibrosis on Ruxolitinib: A Case Report and Review of the Literature on Ruxolitinib‐Associated Invasive Fungal Infections,” Journal of Fungi 10, no. 4 (2024): 264. 38667935 10.3390/jof10040264 PMC11051496

[46] M. V. Dioverti , O. M. Abu Saleh , and A. J. Tande , “Infectious Complications in Patients on Treatment With Ruxolitinib: Case Report and Review of the Literature,” Infectious Diseases 50, no. 5 (2018): 381–387. 29251529 10.1080/23744235.2017.1390248

[47] J. Arnall , L. Lyle , and D. C. Moore , “Advances in Myelofibrosis Management: New Janus Kinase Inhibitors Beyond Ruxolitinib,” Journal of the Advanced Practitioner in Oncology 15, no. 8 (2024): 1–13. 39802534 10.6004/jadpro.2024.15.8.6 PMC11715402

[48] C. N. Harrison , A. M. Vannucchi , C. Recher , et al., “Momelotinib Versus Continued Ruxolitinib or Best Available Therapy in JAK Inhibitor‐Experienced Patients With Myelofibrosis and Anemia: Subgroup Analysis of SIMPLIFY‐2,” Advances in Therapy 41, no. 9 (2024): 3722–3735. 38990433 10.1007/s12325-024-02928-4 PMC11349857

[49] F. Palandri , L. Masarova , S. Verstovsek , et al., “P1062: Indirect Treatment Comparison of Momelotinib vs Fedratinib Safety in Patients With Myelofibrosis,” Hemasphere 7, no. S3 (2023): e303449c. 10.1080/14796694.2025.2511564 PMC12218749 40476514

[50] D. Tremblay , A. King , L. Li , et al., “Risk Factors for Infections and Secondary Malignancies in Patients With a Myeloproliferative Neoplasm Treated With Ruxolitinib: A Dual‐Center, Propensity Score‐Matched Analysis,” Leukemia & Lymphoma 61, no. 3 (2020): 660–667. 31711337 10.1080/10428194.2019.1688323 PMC7771191

