# Supplementary material for: Long‐Term Safety Profile of Ruxolitinib in Chronic Myeloproliferative Neoplasms: A Comprehensive Real‐World Analysis
Source: EJHaem. 2025 Oct 29;6(6):e70152. doi: 10.1002/jha2.70152 (PMC12570962; doi:10.1002/jha2.70152)
Supplement: Supplementary file 1 — Supplementary Table: Comparison of the baseline characteristics of two cohorts before matching: patients with exposure of ≥3 years versus <3years. [file JHA2-6-e70152-s001.docx]

|  | Exposure ≥3y | Exposure <3y | p |
| --- | --- | --- | --- |
| Cohort size, n | 1,368 | 416 |  |
| Mean age (SD), years | 65.6 (12.2) | 65 (12.6) | NS |
| Sex | | | |
| Male, n (%) | 576 (42.1) | 178 (47.5) | NS |
| Female, n (%) | 714 (52.2) | 186 (49.6) | NS |
| Unknown, n (%) | 78 (5.7) | 11 (2.9) | NS |
| MPN | | | |
| Myelofibrosis | 420 (30.7) | 157 (37.7) | <0.05 |
| Polycythemia vera | 616 (45) | 134 (32.2) | <0.05 |
| ET | 332 (24.3) | 125 (30.1) | <0.05 |
| Median FU (IQR), years | 5.8 (3.2) | 4.8 (3.1) | <0.05 |
| Race | | | |
| White, n (%) | 984 (71.9) | 291 (77.6) | NS |
| Black or African American, n (%) | 73 (5.3) | 25 (6.7) | NS |
| Asian, n (%) | 68 (5) | 14 (3.7) | NS |
| Other or unknown, n (%) | 243 (17.8) | 81 (22) | NS |
| Prior hydroxyurea | 556 (41) | 138 (37) | NS |
| Comorbidities | | | |
| Tobacco use, n (%) | 85 (6) | 33 (6) | NS |
| Overweight and obesity, n (%) | 94 (7) | 37 (10) | NS |
| Actinic keratosis, n (%) | 111 (8) | 30 (8) | <0.05 |
| Prior NMSC, n (%) | 110 (8) | 30 (8) | <0.05 |

Supplementary Table. Comparison of the baseline characteristics of two cohorts before matching: patients with exposure of ≥3 years versus <3years
